# Direct Sum Matrix Game with Prisoner's Dilemma and Snowdrift Game

**DOI:** 10.1371/journal.pone.0081855

**Published:** 2013-12-20

**Authors:** Chengzhang Ma, Wei Cao, Wangheng Liu, Rong Gui, Ya Jia

**Affiliations:** 1 Department of Physics and Institute of Biophysics, Huazhong Normal University, Wuhan, China; 2 Department of Applied Physics, College of Science, Huazhong Agricultural University, Wuhan, China; University of Maribor, Slovenia

## Abstract

A direct sum form is proposed for constructing a composite game from two 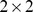 games, prisoner's dilemma and snowdrift game. This kind of direct sum form game is called a multiple roles game. The replicator dynamics of the multiple roles game with will-mixed populations is explored. The dynamical behaviors on square lattice are investigated by numerical simulation. It is found that the dynamical behaviors of population on square lattice depend on the mixing proportion of the two simple games. Mixing SD activities to pure PD population inhibits the proportion of cooperators in PD, and mixing PD activities to pure SD population stimulates the proportion of cooperators in SD. Besides spatial reciprocity, our results show that there are roles reciprocities between different types of individuals.

## Introduction

As mathematical framework, evolutionary game theory has been used to model evolutions in social, economical and biological systems widely [1]–[3]. One of the fundamental problems in this theory is the evolution and maintenance of cooperation among selfish individuals [4]–[8]. Two prominent mathematical metaphors have attracted most attention in theoretical and experimental studies of cooperation, the prisoner's dilemma and the snowdrift games [9]–[11]. In both games, each player decides whether to cooperate or defect. In the prisoner's dilemma(PD), cooperation of the players results to the highest payoff which is equally shared among the two players, yet individual defectors will do better if the opponent decides to cooperate. Since the selfish players want to maximize their own income and they both decide to defect. None of them gets a profit and instead of equally sharing the payoff received by mutual cooperation, they end up almost empty-handed. The snowdrift(SD) game has different payoffs matrix compare with the PD. It is an interesting alternative for the study of cooperation, and individuals in such game can gain access to benefits for the pair at one individual cost. Cooperators have to bear the costs whereas defectors are not. Both games represent social dilemmas [12], in which defectors are prone to exploit cooperators, and have an evolutionary advantage over cooperators in populations. But cooperation is widespread in the real world and required for many levels of biological organization ranging from genes to groups of animals. Cooperation is also the decisive organizing principle of human societies. Therefore, the underlying mechanisms of cooperation are much needed and have been investigated extensively in different contexts, such as kin selection [13], direct reciprocity [4], [9], [14]–[16], indirect reciprocity [17]–[19], group selection [20]–[22] and network reciprocity [23], [24], which are summarized as five rules [25]. The network reciprocity could also be regarded as a generalization of spatial reciprocity.

To study the dynamical behaviors of a spatial game, the following points should be considered: the spatial structure on which the game runs, the interactive ways of individuals, the updating and mutation rules. The most common spatial structure is the square lattice [23], in which the cooperators can form clusters to protect themselves against the exploitation by defectors. Small-world networks, scale-free graphs and evolving networks may also serve as the spatial structures of a game [26]. By adopting coevolutionary rules, coevolutionary games can rearrange network structures [27]. For instance, Chen et al. [28] have proposed a coevolutionary rule in spatial public goods games. The interactive ways are determined by payoff functions, which are known as payoff matrices in matrix games, and the scope of opponents, such as Von Neumann and Moore neighborhood in square lattice, pairwise interaction, group interaction, etc. Strategy updating and mutation rules may also affect evolutionary dynamics. Some examples include the birth-death and imitation rule [29], the proportional imitation rule [30], the reinforcement learning adoption rule [31], or the Fermi rule [32]. Wang et al. [33] have recently considered an adaptive strategy-adoption rule in which the focal player evaluates its strategy by comparing the average payoff of each strategy in the neighborhood. They have shown that the survivability of cooperators has a significant increment in contrast with that of pairwise strategy updating. Based on win-stay-lose-shift rule, Liu et al. [34] introduced a win-stay-lose-learn strategy updating rule in spatial prisoner's dilemma game, where the focal player attempts to update her strategy only when her payoff is less than her aspiration.

Most previous works about PD and SD game have been referred to the consideration of the individual interaction being one fold, which means that all interactions adopt either PD or SD payoff matrix [32]–[35]. That is all individuals participate in the same kind of game. However, the situation that individuals involved in multiple activities is happened frequently in economic and social activities or biological behaviors. In the context of game theory, individuals participate in multiple games and act different roles in these games. Such games can be called *multiple roles game*(MRG).

In the present work, a simple MRG model with PD and SG payoff matrix is constructed. In the modeling approach section, we describe the model for multiple roles games with prisoner's dilemma and snowdrift game. In the results section, the replicator dynamics of the multiple roles game with will-mixed populations is explored, and the dynamical behaviors on square lattice are calculated in a numerical way over the parameter space. In the last section, conclusions are presented and the potential clues to other general cases are predicated.

## Modeling Approach

To elaborate the idea of MRG, let's focus on two-strategy games. The two strategies can be denoted as cooperation and defection, abbreviated as 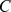 and 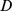, respectively. The payoff matrix has the form
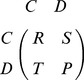
(1)Here, a player with 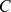 strategy obtains profit 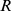 from another 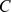 player, 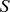 from a 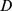 player. Similarly, a 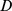 player obtains 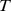 from a 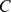 player, 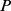 from another 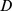 player. The Prisoner's dilemma(PD) game starts on the condition as following:
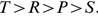
(2)The relationship 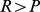 implies that mutual cooperation is superior to mutual defection, while the relationships 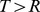 and 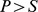 imply that defection is the dominant strategy regardless of opponent's strategy. The SD game has a different condition as shown below:
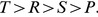
(3)In contrast to PD, the best strategy now depends on the opponent's strategy. The relationship 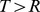 implies that cooperation is better if the opponent defects, while 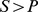 implies that defection is better if the opponent cooperates.

The general payoff matrix form(1) has too many parameters to be analyzed. To prevent this kind of situation, the payoff matrix form with two parameters is given for two-strategy game as following [36], [37],
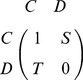
(4)with 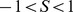 and 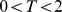. If 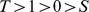, (4) indicates PD payoff matrix, and if 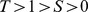, it indicates SD payoff matrix. If a MRG model with PD and SD is constructed with the payoff matrix (4), it would have four parameters which will lead to complexity of analysis. Therefore, it is necessary to construct single parameter payoff matrices of PD and SD. One can rescale the payoff matrix (4) to single parameter forms [10], [11], [35], [38] as shown in Eq.(5)

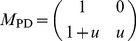
(5)for the PD game, and
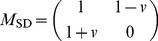
(6)for the SD game. Both 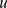 and 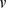 are constrained to the interval 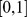. Obviously, (5) and (6) satisfy the corresponding inequalities (2) and (3) respectively.

**Figure 1:**
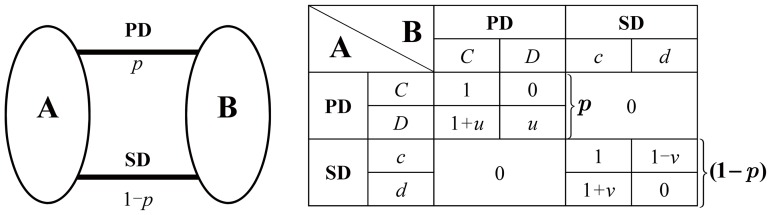
Multiple roles game(MRG) model with PD and SD. There are two interaction channels between individual A and B. The channel PD has a probability 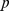 to be selected and the channel SD has a probability 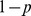 to be selected.

Now consider a population where each individual has two identities: prisoner for PD game and driver for SD game. Each individual participates in two games simultaneously. When two individuals interplay, the process is divided into two steps. In the first step, they have to determine what kind of game they will play, and in the second step, they play the selected game according to their own strategy. Let us assume that in a pairwise interplay they choose PD game with probability 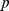, SD game with probability 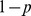. In this way, a simply MRG model is constructed(Fig. 1). The left panel of Fig. 1 shows that there are two channels of interaction between the individual A and B, the PD channel with probability 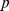 and the SD channel with probability 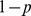. The right panel of Fig. 1 shows the details of the payoff matrix of MRG. Here we denote strategies with capital letters 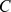 and 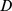 in PD game, and lowercase letters 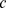 and 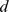 in SD game. Formally, an extended payoff matrix 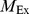 can be obtained by direct sum for the MRG,
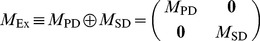
(7)


Since it is impossible that an individual who opts for PD game interacts with the individual who has made a choice of SD game with 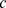 or 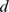 strategy, we express these cases with two sub-matrices **0** in (7).

To construct a spatial model of MRG, let's place each individual on one cell in a two-dimensional 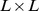 square lattice with periodic boundary conditions being used. There are no empty cells on lattice. An individual interacts with its Moore neighborhoods, and its total payoff is the sum of payoffs obtained from its all neighbors. The population is asynchronously updated by the rule known as the *replicator rule* or *proportional imitation rule* [30], [36], [39]. Randomly selecting a focal individual 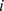, let 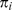 denotes its total payoff, 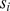 its category, or its strategy set. Individual 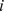 updates its strategy set by comparing its total payoff 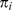 to the total payoff 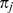 of a randomly selected neighbor 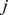. The focal individual 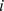 adopts 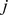
's strategy set 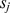 with a probability 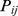 proportional to the payoff difference, provided that 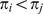. So the probability 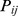 is given by
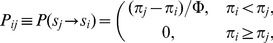
(8)where 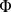 denotes a normalization constant to ensure 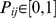. Here
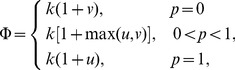
(9)where 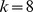 represents the number of 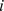
's Moore neighbors. After updating its strategy set, the payoff of the focal individual 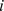 with that of its all neighbors are reset to zero. Note that the current MRG model does not take into account mutations in updating strategy sets, and individuals propagate every strategy of its each role to the next generation perfectly.

## Results

### Replicator dynamics of the multiple roles game

According to the strategies individuals adopt, they can be divided into four categories, which can be recorded as 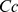, 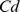, 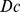, 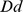. With this convention, 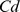 means that the individual adopts strategy of cooperation in PD games, and defection in SD games, and the other three categories are similar to 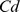. In the replicator dynamics, 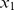, 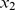, 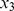, 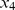 represent the fractions of 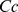, 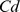, 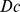, 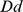 respectively. Without arising confusions, sometimes 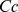, 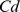, 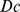, 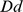 also represent the fractions of the corresponding types of the individuals. The payoff matrix for the four strategies is:
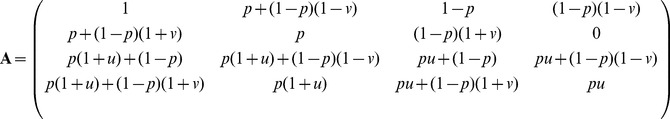
(10)


The deterministic evolutionary game dynamics are given by the replicator equation [40], [41] 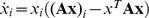. Here 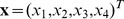, and 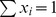. Let 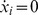, it is not difficult to get eight equilibrium points: 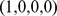, 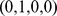, 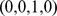, 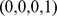, 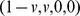, 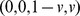, 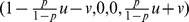, 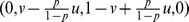. Apparently, only the last two are related to the parameter 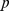. Consideration of the constraint 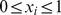, the seventh and eighth are only valid as they meet criterion 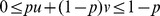 and 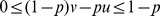, respectively. It shows that the mixed probability 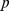 of PD and SD can influence the number of the equilibrium points.

### Effect of the mixed probability 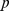 on population structure

**Figure 2:**
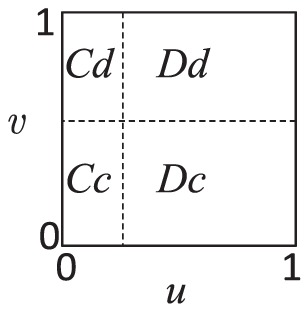
Schematic presentation of 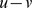 parameter plane. Simulation runs with the parameters as 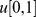 step 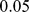, 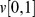 step 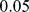.

**Figure 3:**
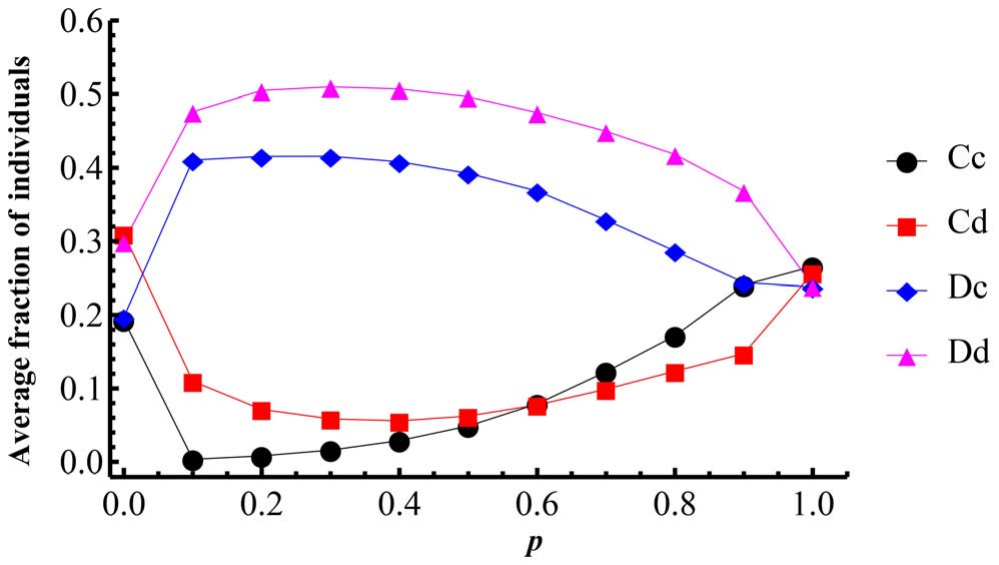
The average fractions over 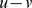 parameter plane of 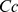, 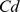, 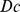, 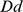 in dependence on 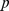. For each 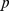, the simulations run on 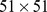 square lattice with 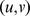 in discrete 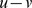 parameter plane. The initial average fractions of the four types are all 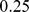.

Simulation runs on a 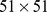 lattice with four categories individuals initial randomly uniform distributing over it equiprobably. To ensure a correct convergence, 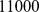 time steps are employed [36], where one time step means each individual updates its strategies one time averagely. Preliminary simulation results shows that, for 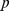 from 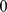 to 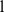 step 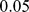, the two-dimensional 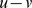 parameter plane can be divided into four areas roughly as shown in Fig. 2. As expected, individuals tend to cooperate for smaller 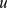 or 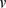 values in both games, to defect for larger 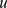 or 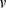 values. As a result, 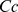 individuals locate mainly in left bottom region on 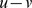 plane, 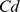 in left up region, 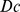 in right bottom region, and 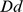 in right up region. For more details, we set the parameter 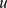 at the interval 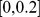 with step 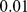, 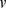 at 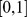 with step 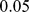, and for 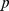 from 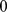 to 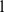 step 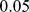. For each 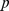, we introduce average fractions of 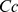, 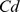, 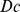, 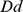 over the region 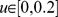 and 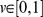 in the 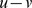 phase plane. These global indexes fall in the range 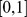. Fig. 3 shows the simulation results of the influence of 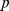 on the average fractions of the four categories of individuals. The left endpoints of the curves correspond to the case 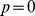, which indicate the population structure with pure SD game. The right endpoints of the curves correspond to the case 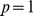, which indicate the population structure with pure PD game. Comparing with these reference endpoints the average fractions of 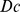 and 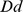 form two convex curves, and the average fractions of 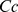 and 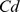 form two concave curves. It is quite obvious that from the global perspective, mixing SD and PD games will stimulate 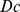 and 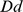, and inhibit 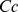 and 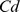 individuals.

**Figure 4:**
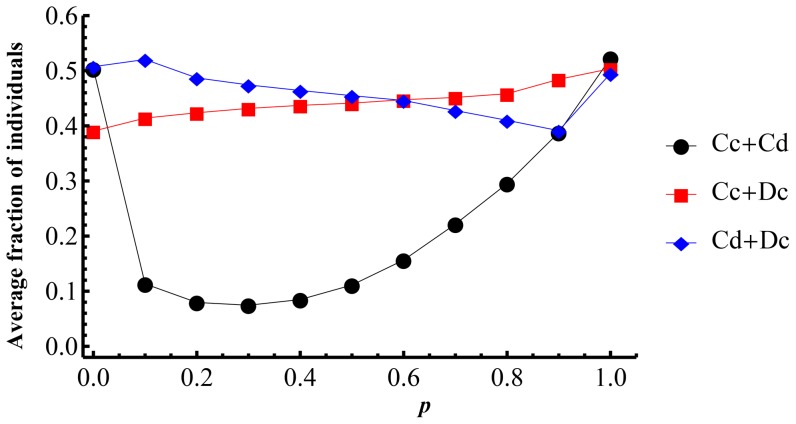
The average fractions of 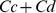 as PD C, 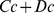 as SD c, and 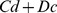 in dependence on 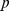. For each 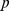, the simulations run on 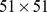 square lattice with 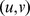 in discrete 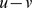 parameter plane. The initial average fractions of the four types are all 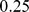.

**Figure 5:**
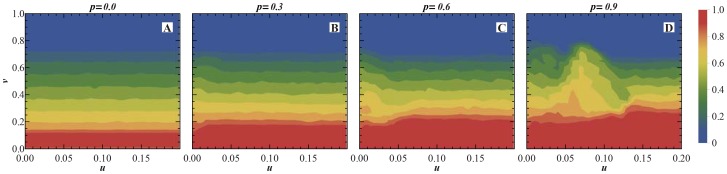
The fractions of 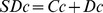 individuals in 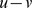 parameter plane with p = 0.0(pure SD population), 0.3, 0.6, 0.9. The initial average fractions of 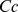, 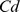, 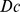, 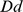 are all 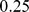 and the individuals are randomly distributed on lattices evenly in the beginning.

**Figure 6:**
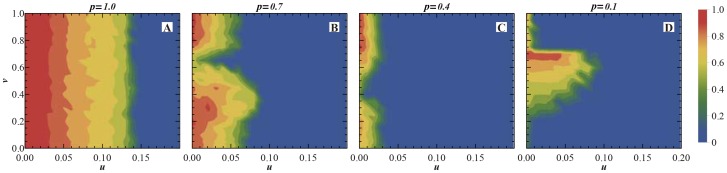
The fractions of 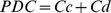 individuals in 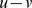 parameter plane with 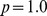
(pure PD population), 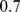, 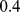, 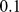. The initial fractions of 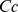, 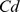, 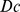, 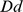 are all 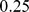 and the individuals are randomly distributed on lattices evenly in the beginning.

**Figure 7:**
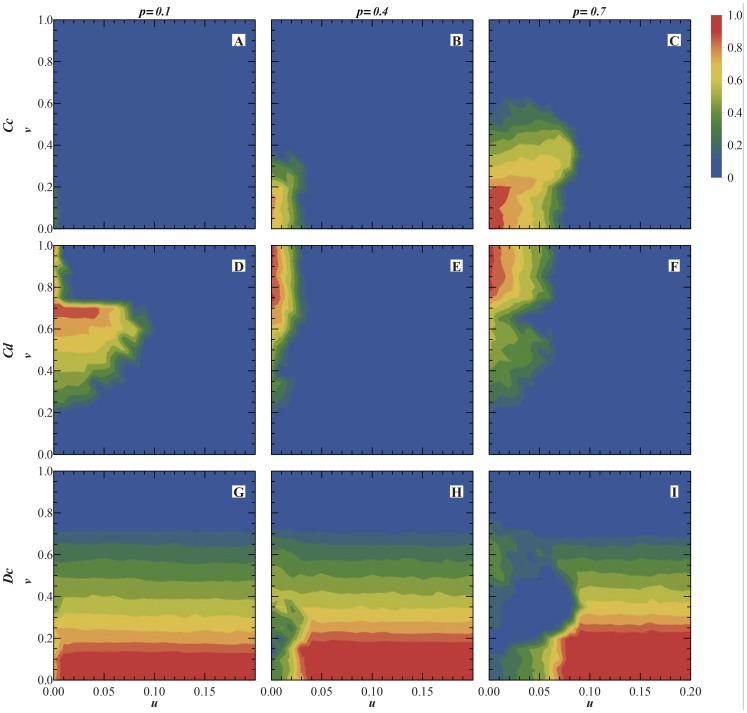
The fractions of 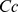 (the first row), 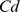 (the second row) and 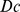 (the third row) individuals in 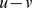 parameter plane. For the first column p = 0.1; the second column p = 0.4;the third column p = 0.7.

For comparison with the pure PD and SD games, let 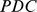 represents the average fraction of players with 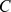 strategy in PD games, which equals to 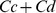, and 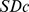 represents the average fraction of players with 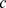 strategy in SD games, which equals to 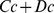. The average fractions of 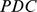 and 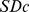 are given in Fig. 4 as functions of 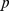. The 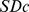 curve increases with the increasing mixed probability 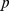, which indicates cooperators of SD increase as 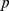 increases. It means that mixing PD game in SD population can stimulate cooperate fraction in SD. The average fraction of 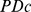 forms a concave curve and stills bottoming out in the area 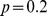 to 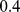. From the view of PD, mixing SD game in PD population tends to inhibit the cooperate 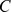 strategy in PD game. The extent of inhibition reaches a maximum at 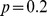 to 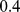. The population fractions distributing in the phase plane can reveal more details of the evolutionary dynamics. Fig. 5 shows the fraction of 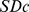 distributing in 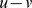 parameter plane. As a frame of reference, Fig. 5A(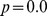) represents the case of pure SD population, in which the 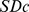 individuals are mainly distributed in the low value zone of 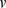, or the bottom of 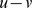 plane, and have no association with the parameter 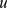. Fig. 5B,C,D(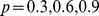 respectively) show that with the increase of 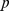, the 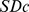 region expands slowly to the zone of the larger 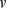 value. This is in consistency with the 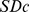 curve in Fig. 4. Fig. 6 shows the the fraction of 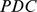 distributing in 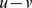 plane. As a frame of reference, Fig. 6A(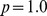) represents the case of pure PD population, in which the 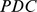 individuals are mainly distributed in the low value zone of 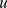, or the left of 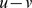 plane, and have no association with the parameter 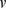. Fig. 6B,C(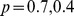 respectively) show that with the decrease of 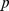, the 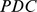 region compresses to the zone of the smaller 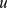 value inhomogeneously. But one should note that for 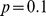
(Fig. 6 D), the 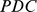 region has an anomalous expansion comparing with that of 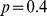
(Fig. 6 C). This kind of anomalous expansion is consistent with the curve 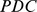 in Fig. 4 which decreases first and then increases with 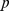 from 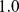 to 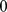. To explore the origin of the 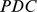 anomalous expansion at lower 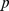 values, it should be noted that 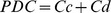. The fractions of 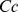 and 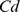 in 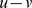 parameter plane are shown in Fig. 7 A–F for several different 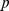 values. From Fig. 7 A,D we know that the anomalous expansion of 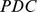 for 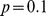 is originated from that of 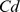.

It is well known that if there is only one kind of game in population, such as PD or SD game, there exists effect of spatial reciprocity in lattice space [23], [24]. But if an individual in lattice space is drawn into multiple games, its different strategies in each game may lead up to the situation: what one loses on the swings, she gets back on the roundabouts. In the current situation, it becomes that what one loses as a prisoner in PD, she gets back as a driver in SD, or vice verse. This phenomenon can be called as *roles reciprocity*. This effect superimposes on that of spatial reciprocity to influence the fractions of individuals on payoff matrix parameter space 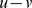 plane.

In the PD and SD MRG case, from comparing the fractions of 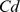 and 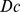 (Fig. 7 D–F, G–I) in 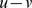 parameter plane, it is found that the regions of 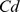 and 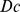 extend to the side of each other. This phenomenon is a typical demonstration of *roles reciprocity*. Roles reciprocity strengthens the survival ability of 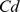 and 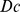 players. Although individuals with strategies 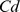 or 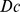 have no maximal fractions in four categories(Fig. 3), they have more tolerance in unfavorable parameters(Fig. 4 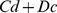 curve) in appropriate parameter intervals of 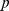, 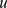 and 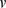.

**Figure 8:**
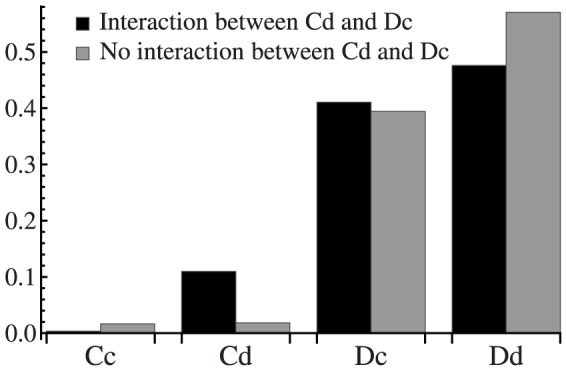
Illustration of the effect of roles reciprocity of 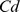 and 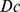 with 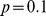. Without the interaction between 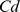 and 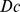, their average fractions descend obviously.

**Figure 9:**
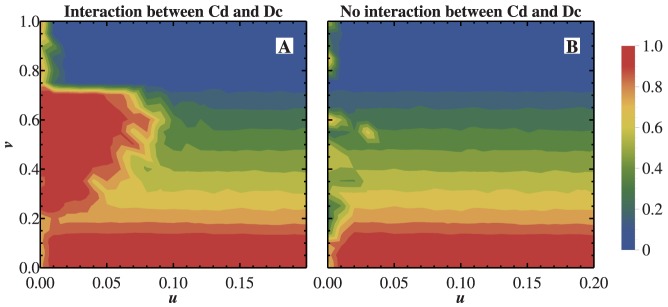
Illustration of the effect of roles reciprocity of 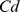 and 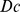 with 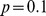 in 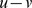 parameter plane. A) The fraction of 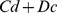 with the interaction between 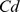 and 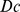. The cross domain of 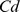 and 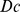 obviously exists. B) The fraction of 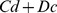 without the interaction between 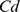 and 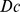. The cross domain of 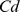 and 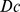 vanishes completely.

To illustrate the effect of roles reciprocity, we cancel the interaction between the 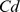 and 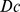 individuals for simulation with 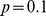. Comparing with roles reciprocity(the case of existing interaction between the 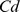 and 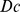), the average fractions of 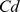 and 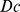 descend in the absence of interaction between them(Fig. 8). In 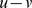 parameter plane, the effect of roles reciprocity become ever more evident(Fig. 9). Without the roles reciprocity, the cross domain of 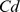 and 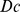 vanished completely(Fig. 9 B). It can be say that 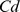 and 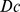 individuals establish a ecological chain in a small parameter region with about 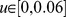 and 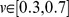 at 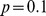. The fact that the region is too small reflects the frangibility of that toy ecosystem.

## Discussion

In the real world, it is common that an agent acts as multiple identities. It can be say that the agents participate in multiple games and have multiple roles, and it can be called multiple roles game, or MRG. To imitate these cases, a evolutionary game model is introduced, in which each agent has two identities, one for PD game and another for SD game. A parameter 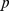 is introduced to indicate the probability to select PD game for each pair interaction. It shows that the mixed probability 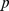 of PD and SD can influence the number of the equilibrium points in the deterministic evolutionary game dynamics. In the spatial MRG, the agents are placed on patches in a two-dimensional lattice with periodic boundary conditions being used. The number simulation shows that mixing SD and PD games will stimulate 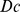 and 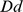, and inhibit 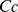 and 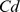 individuals from the global perspective. Comparison with the pure PD or SD games shows that SD cooperators ratio(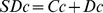) increases as 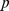 increase, and PD cooperators ratio(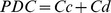) is inhibited for 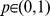.

The population fraction distributions in the 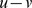 phase plane reveals that besides spatial reciprocity, there exist roles reciprocity, which means for some kind of agents their multiple identities will have help them to obtain benefits in population. In the MRG model with PD and SD games, because of the roles reciprocity, 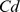 and 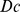 individuals form a ecological chain in appropriate parameter region. These toy ecosystems are fragile on account of too small of the appropriate parameter areas.

To configure our multiple roles game model, we introduced direct sum form to construct the payoff matrix from simple game's payoff matrix. This method gives a way to construct complex MRG model from simple sub games. In game theory experiments, participators often influence by various factors. Or in biology, a group may have more than one function. If one need to construct a game theory frame in these cases, the MRG modeling method could be an option to consider.

## References

[1] Smith J (1982) Evolution and the Theory of Games. Cambridge University Press, Cambridge,UK.

[2] Colman A (1995) Game theory and its applications in the social and biological sciences. Butterworth-Heinemann, Oxford, UK.

[3] Nowak M (2006) Evolutionary dynamics: exploring the equations of life. Harvard University Press, Cambridge, MA, USA.

[4] Trivers R (1971) The evolution of reciprocal altruism. Q Rev Biol 46: 35–57.

[5] Lotem A, Fishman M, Stone L (1999) Evolution of cooperation between individuals. Nature 400: 226–227. 10.1038/22247 10421362

[6] Boyd R, Gintis H, Bowles S, Richerson P (2003) The evolution of altruistic punishment. Proc Nat Acad Sci USA 100: 3531–3535. 12631700 10.1073/pnas.0630443100 PMC152327

[7] Wang J, Fu F, Wu T, Wang L (2009) Emergence of social cooperation in threshold public goods games with collective risk. Phys Rev E 80: 016101. 10.1103/PhysRevE.80.016101 19658768

[8] Szolnoki A, Perc M (2012) Conditional strategies and the evolution of cooperation in spatial public goods games. Phys Rev E 85: 026104. 10.1103/PhysRevE.85.026104 22463276

[9] Axelrod R, Hamilton W (1981) The evolution of cooperation. Science 211: 1390–1396. 7466396 10.1126/science.7466396

[10] Hauert C, Doebeli M (2004) Spatial structure often inhibits the evolution of cooperation in the snowdrift game. Nature 428: 643–646. 15074318 10.1038/nature02360

[11] Doebeli M, Hauert C (2005) Models of cooperation based on the prisoner's dilemma and the snowdrift game. Ecol Lett 8: 748–766.

[12] Kerr NL (1983) Motivation losses in small groups: A social dilemma analysis. J Pers Soc Psychol 45: 819–828.

[13] Hamilton WD (1964) The genetical evolution of social behaviour I. J Theor Biol 7: 1–16. 5875341 10.1016/0022-5193(64)90038-4

[14] Axelrod R (1984) Evolution and the Theory of Games. New York: Basic Books.

[15] Nowak MA, Sigmund K (1992) Tit for tat in heterogeneous populations. Nature 355: 250–253.

[16] Nowak M, Sigmund K (1993) A strategy of win-stay, lose-shift that outperforms tit-for-tat in the prisoner's dilemma game. Nature 364: 56–58. 8316296 10.1038/364056a0

[17] Nowak MA, Sigmund K (1998) Evolution of indirect reciprocity by image scoring. Nature 393: 573–577. 9634232 10.1038/31225

[18] Nowak MA, Sigmund K (2005) Evolution of indirect reciprocity. Nature 437: 1291–1298. 16251955 10.1038/nature04131

[19] Ohtsuki H, Iwasa Y (2006) The leading eight: social norms that can maintain cooperation by indirect reciprocity. J Theor Biol 239: 435–444. 16174521 10.1016/j.jtbi.2005.08.008

[20] Wilson DS (1975) A theory of group selection. Proc Nat Acad Sci USA 72: 143–146. 1054490 10.1073/pnas.72.1.143 PMC432258

[21] Wilson DS (1983) The group selection controversy: history and current status. Annu Rev Ecol Syst 14: 159–187.

[22] Traulsen A, Nowak MA (2006) Evolution of cooperation by multilevel selection. Proc Nat Acad Sci USA 103: 10952–10955. 16829575 10.1073/pnas.0602530103 PMC1544155

[23] Nowak M, May R (1992) Evolutionary games and spatial chaos. Nature 359: 826–829.

[24] Ohtsuki H, Hauert C, Lieberman E, Nowak M (2006) A simple rule for the evolution of cooperation on graphs and social networks. Nature 441: 502–505. 16724065 10.1038/nature04605 PMC2430087

[25] Nowak M (2006) Five rules for the evolution of cooperation. Science 314: 1560–1563. 17158317 10.1126/science.1133755 PMC3279745

[26] Szabó G, Fáth G (2007) Evolutionary games on graphs. Physics Reports 446: 97–216.

[27] Perc M, Szolnoki A (2010) Coevolutionary games-a mini review. BioSystems 99: 109–125. 19837129 10.1016/j.biosystems.2009.10.003

[28] Chen X, Liu Y, Zhou Y, Wang L, Perc M (2012) Adaptive and bounded investment returns promote cooperation in spatial public goods games. PloS one 7: e36895. 22615836 10.1371/journal.pone.0036895 PMC3353963

[29] Ohtsuki H, Nowak MA (2006) Evolutionary games on cycles. Proc R Soc Lond B 273: 2249–2256. 10.1098/rspb.2006.3576 PMC1635521 16901846

[30] Schlag K (1998) Why imitate, and if so, how?: A boundedly rational approach to multi-armed bandits. J Econ Theory 78: 130–156.

[31] Wang S, Szalay MS, Zhang C, Csermely P (2008) Learning and innovative elements of strategy adoption rules expand cooperative network topologies. PLoS One 3: e1917. 18398453 10.1371/journal.pone.0001917 PMC2275790

[32] Szabó G, Töke C (1998) Evolutionary prisoners dilemma game on a square lattice. Phys Rev E 58: 69–73.

[33] Wang X, Perc M, Liu Y, Chen X, Wang L (2012) Beyond pairwise strategy updating in the prisoner's dilemma game. Sci Rep 2: 740–740. 23074647 10.1038/srep00740 PMC3472391

[34] Liu Y, Chen X, Zhang L, Wang L, Perc M (2012) Win-stay-lose-learn promotes cooperation in the spatial prisoner's dilemma game. PloS one 7: e30689. 22363470 10.1371/journal.pone.0030689 PMC3281853

[35] Fu F, Nowak M, Hauert C (2010) Invasion and expansion of cooperators in lattice populations: Prisoner's dilemma vs. snowdrift games. J Theor Biol 266: 358–366. 20619271 10.1016/j.jtbi.2010.06.042 PMC2927800

[36] Roca C, Cuesta J, Sánchez A (2009) Effect of spatial structure on the evolution of cooperation. Phys Rev E 80: 046106. 10.1103/PhysRevE.80.046106 19905389

[37] Roca C, Cuesta J, Sánchez A (2009) Evolutionary game theory: Temporal and spatial effects beyond replicator dynamics. Phys Life Rev 6: 208–249. 20416850 10.1016/j.plrev.2009.08.001

[38] Langer P, Nowak M, Hauert C (2008) Spatial invasion of cooperation. J Theor Biol 250: 634–641. 18068731 10.1016/j.jtbi.2007.11.002 PMC2289997

[39] Helbing D (1992) Interrelations between stochastic equations for systems with pair interactions. Physica A 181: 29–52.

[40] Taylor P, Jonker L (1978) Evolutionary stable strategies and game dynamics. Math Biosci 40: 145–156.

[41] Schuster P, Sigmund K (1983) Replicator dynamics. J Theor Biol 100: 533–538.

